# Case of Wound of the Parietes of the Abdomen, with Protrusion of a Portion of Intestine, Successfully Treated

**Published:** 1825-12

**Authors:** William Dix


Art. V.—*Case of Wound of the Parietes of the Abdomen, with Protrusion of a Portion of Intestine, successfully treated.* By William Dix, Esq.

About six o'clock p.m. on the 18th of August last, a young man, nineteen years of age, was attacked by a bull, and received a wound from the horn, of about three inches, on the left side of the abdomen, midway between the spine of the ilium and inferior margin of the ribs, inclining anteriorly. Nearly three feet of intestines protruded, with a portion of mesentery and omentum. I found him in the field about twenty minutes after the accident: he was very pale; his pulse was rapid, and hardly perceptible; and he was vomiting. A small quantity of blood was mixed with the contents of the stomach. I had him conveyed home, and immediately returned the mesentery and intestines; but, as the omentum was considerably lacerated, I cut the injured parts off. A little hemorrhage succeeded, which was soon suppressed without a ligature. As there was a strong disposition in the parts returned, to protrude, I made one suture, and closed the remaining parts of the wound with adhesive plaster, over which was applied a compress and bandage. I ordered him to have some warm gruel and an anodyne; and to be kept in a horizontal position, and quiet.

19th, in the morning.—Found he had had a tolerable night. Countenance very florid; skin hot; pulse 125, and hard; abdomen very tender; breathing short, with difficulty in swallowing; no sickness.—Venesection to syncope. A common enema to be injected, and a saline aperient taken.

Visited him in the evening.—The injection had procured him a slight evacuation. Pulse and other symptoms the same as the morning.—Bled from the arm again, until syncope was produced.

20th, in the morning.—Has had a pretty good night; pulse 100; no sickness; abdomen tender on pressure.—Twenty leeches to the belly. Capiat ol. ricini ℥ss. omni horâ donec alvus responderit. Injections to be continued.

21st.—Has had six copious evacuations, and is much relieved. Pulse 95.

22d.—Three evacuations from the ol. ricini. Pulse 90. A good deal of pain in the night.—Twenty leeches to the left hypochondrium.

23d.—Pulse 80; bowels open; appetite good.—Diet, gruel.

24th.—Pulse 65. Looked at the wound, which was fast healing: cut out the suture.—Diet, milk and broth. To take castor-oil when necessary, and to be kept in bed.

From this time he went on progressively recovering, and is now perfectly well. In less than a fortnight after the accident, he walked about his master's fields, but which was contrary to my advice; and he was near paying dearly for his temerity, for he caught cold, and was attacked with symptoms of peritoneal inflammation, which, however, were speedily subdued by copious bleeding from the arm, and twenty leeches to the abdomen.

*Long Buckby, Northamptonshire;*
*Sept.* 18*th,* 1825.

